# A Deep Reinforcement Learning-Optimized Blood Flow Profile for Enhanced Oxygenation Efficiency in Membrane Oxygenators

**DOI:** 10.3390/membranes16010004

**Published:** 2025-12-23

**Authors:** Junwen Yu, Yuan Liu, Huaiyuan Guo, Qingyang Cheng, Junlong Meng, Ming Yang

**Affiliations:** College of Automation and Intelligent Sensing, Shanghai Jiao Tong University, Shanghai 200240, China

**Keywords:** membrane oxygenator, deep reinforcement learning, gas exchange efficiency, extracorporeal life support

## Abstract

The membrane oxygenator serves as the core component of extracorporeal life support systems, and its gas exchange efficiency critically influences clinical outcomes. However, gas transfer is predominantly limited by the diffusion barrier within the blood-side boundary layer, where saturated red blood cells accumulate. Current research focuses mainly on static approaches such as optimizing fiber bundle configuration to promote passive blood mixing or modifying material properties, which are fixed after fabrication. In contrast, dynamic blood flow control remains an underexplored avenue for enhancing oxygenator performance. This study proposes an active pulsatile flow control method that disrupts the boundary layer barrier by optimizing periodic flow profiles, thereby directly improving gas exchange. A deep reinforcement learning framework integrating proximal policy optimization and long short-term memory networks was developed to autonomously search for optimal flow waveforms under constant flow conditions. A simplified stacked-plate membrane oxygenator was specially designed as the experimental platform to minimize flow path interference. Experimental results demonstrate that the optimized pulsatile profile increases the oxygen transfer rate by 20.64% without compromising hemocompatibility.

## 1. Introduction

Extracorporeal Membrane Oxygenation (ECMO) is an extracorporeal life support technology used for cardiogenic shock, for severe acute respiratory failure, and as a bridge to lung transplantation for end-stage lung disease, providing critical life support for critically ill patients [1]. This system can rapidly improve patient oxygenation, achieve protective lung ventilation, interrupt hypoxia-mediated tissue damage, and effectively alleviate refractory hypoxemia, inadequate tissue perfusion, or hypercapnia [2,3].

A traditional ECMO system primarily consists of core components including a blood pump (artificial heart), an oxygenator (artificial lung), tubing and cannulas, a monitoring system, an anticoagulation management system, and a heat exchanger [4,5]. The blood pump is responsible for driving blood flow, while the oxygenator performs gas exchange. Hollow fiber membranes (HFMs) serve as essential components in oxygenators, enabling efficient gas exchange while maintaining effective separation between blood and gas phases [6]. While gas transport across the hollow-fiber membrane (HFM) itself occurs primarily by diffusion, the dominant resistance to mass transfer in membrane oxygenators lies within the blood-side boundary layer, where saturated red blood cells accumulate and create a significant diffusional barrier [7]. This layer significantly restricts the transport of oxygen and carbon dioxide, thereby limiting the overall efficiency of gas exchange. As the boundary layer constitutes the dominant resistance to gas exchange, the overall efficiency is reduced, necessitating a larger gas-exchange surface area to compensate for the limited performance. This requirement leads to increased device size and complex circuit configurations, thereby substantially expanding the interfacial contact between blood and artificial material surfaces. Prolonged exposure under such conditions can induce hemolysis, inflammatory responses, platelet activation, and thrombotic risks [8,9,10,11,12]. Hence, maximizing oxygenation efficiency under severe spatial constraints remains a critical objective in oxygenator development.

Currently, research on performance optimization of oxygenators primarily revolves around two main directions: structural design and material innovation. In the field of structural design, Martins Costa et al. [13] optimized the arrangement of hollow fiber tubes, effectively enhancing oxygenation efficiency. Focke et al. [14] combined three-dimensional numerical simulations with blood experiments to analyze the impact of fiber arrangement on gas exchange, discovering that a circumferential arrangement could achieve twice the oxygenation efficiency of a longitudinal arrangement, albeit with limitations in processing complexity and operational adaptability. Based on computational fluid dynamics (CFD) simulations, Fu et al. [15] proposed that a multi-inlet/outlet design helps optimize blood flow distribution and reduces thrombosis risk. In the domain of material innovation, researchers have developed various advanced membrane materials that significantly enhance gas permeability and hemocompatibility [16,17,18,19,20].

However, these passive approaches become fixed upon manufacturing and lack the adaptability to respond to varying physiological conditions during clinical use. In contrast, actively modulating blood flow patterns to enhance blood mixing and oxygenation efficiency remains a relatively unexplored area of research. The core mechanism involves the precise control of pump parameters to regulate blood residence time and boundary layer thickness within the oxygenator. This effectively reduces resistance to transmembrane gas exchange, thereby maximizing oxygenation efficiency under constrained membrane surface area. The potential advantages of this approach are twofold. First, flow field optimization can enhance hemodynamic energy, promoting perfusion to distal organs [21]. Second, by improving flow uniformity, it helps eliminate flow stagnation zones, consequently inhibiting thrombus formation—an effect recently validated by experimental studies [22]. It must be emphasized that flow control strategies, such as introducing pulsatility, require careful evaluation, as they may alter shear stress distributions and potentially increase the risk of damage to blood components.

Despite this, research on how specific blood flow profiles influence oxygenation efficiency remains significantly constrained. The limited exploration of active dynamic blood flow control likely stems from three interconnected challenges: the intrinsic difficulty of modeling highly complex and nonlinear hemodynamic systems [23], the necessity for control strategies that can safely balance multiple competing objectives (such as maximizing gas exchange while minimizing hemolysis and thrombosis), and the current technological limitations of existing pulsatile flow devices [24,25,26], which struggle to generate precisely controlled, pulsatile waveforms reliably. Consequently, most current studies are primarily confined to the sinusoidal pulsatile flow profile, focusing on parameters like frequency and amplitude, yet findings often yield inconsistent conclusions due to variations in oxygenator internals such as fiber arrangement and flow resistance [27,28]. While Schraven et al. [29] examined a sinusoidal flow profile, their work was constrained by the experimental setup, which limited the generation and analysis to approximate sinusoidal waveforms and precluded the investigation of more complex flow patterns. Moreover, the potential differential effects of various waveforms on oxygenation efficiency were likely obscured in their study. This limitation arises because the coiled fiber oxygenator they employed not only introduces substantial geometric damping that distorts the fidelity of input waveforms, but also provides inherent, strong passive mixing—both of which can mask the distinct effects that different flow profiles might exert on gas exchange. Microfluidic approaches—employing engineered microchannels and thin membranes—have demonstrated high gas transfer efficiencies by optimizing flow distribution, but they introduce critical drawbacks including high pressure drops and manufacturing inconsistencies that hinder scalability and broader application [30]. Orizondo et al. [31] developed a fiber oscillation technology that achieved approximately a 40% enhancement in oxygenation, though accompanied by a significant increase in system complexity.

To overcome the limitations of existing research on dynamic flow control, this paper introduces a performance enhancement strategy centered on dynamic flow profile optimization, implemented through a Deep Reinforcement Learning (DRL) framework. Unlike traditional heuristic methods, which often rely on predefined rules, the DRL agent learns to autonomously discover optimal flow profiles through a trial-and-error process driven by environmental interactions [32,33,34]. This data-driven approach demonstrates a strong capability to handle the nonlinear and multi-objective nature of complex fluid dynamics [35]. To validate the effectiveness of this method, this study systematically compares the DRL-optimized profile with the standard half-wave sinusoidal flow, aiming to reveal the influence mechanisms of different dynamic flow profiles on oxygenation efficiency and blood damage, thereby providing a new pathway to break through oxygenation performance limits under constrained membrane area.

## 2. Materials and Methods

### 2.1. Fabrication of the Stacked Membrane Oxygenator

To accurately elucidate the intrinsic effect of blood flow profiles on oxygenation performance independent of geometric confounding factors, this study utilized a custom-designed stacked-plate membrane oxygenator with an optimized flow path. This configuration fundamentally differs from conventional coiled-fiber oxygenators, which typically exhibit a highly tortuous, three-dimensionally complex flow network that induces intense passive mixing, secondary flows, and localized vortices. In such devices, the dominant influence of geometry often overshadows the specific contributions of the flow waveform, making it difficult to isolate the effect of dynamic flow modulation [29]. In contrast, the stacked-plate design employed here provides a streamlined, spatially regular flow path with minimal curvature and well-defined channel boundaries. This structural simplicity intentionally suppresses geometry-dominated mixing mechanisms, thereby allowing the effects of flow profile variations. Additionally, its planar wall structure helps reduce flow stagnation zones caused by blood impingement, thereby mitigating thrombosis risk [36].

The core component of the oxygenator consists of hollow fiber membranes made of poly(4-methyl-1-pentene) (PMP). During fabrication, the hollow fibers were stacked layer-by-layer at an interlayer angle of $24^{\circ }$ and fixed, following the optimized method referenced from [14]. This stacking configuration aims to enhance turbulent mixing by increasing the effective flow path length and introducing moderate flow tortuosity within a limited space, thereby improving gas–liquid contact area and mass transfer efficiency. After stacking, the fiber bundle was potted and cured with a two-component epoxy resin to ensure structural integrity. The cured module was then precision-trimmed using Computerized Numerical Control machining to guarantee dimensional accuracy and consistency. Finally, the housing was fabricated using biocompatible transparent material via 3D printing, and the oxygenator was sealed with epoxy adhesive. The entire process emphasizes minimizing potential interference from complex flow path geometry on performance evaluation.

**Figure 1:**
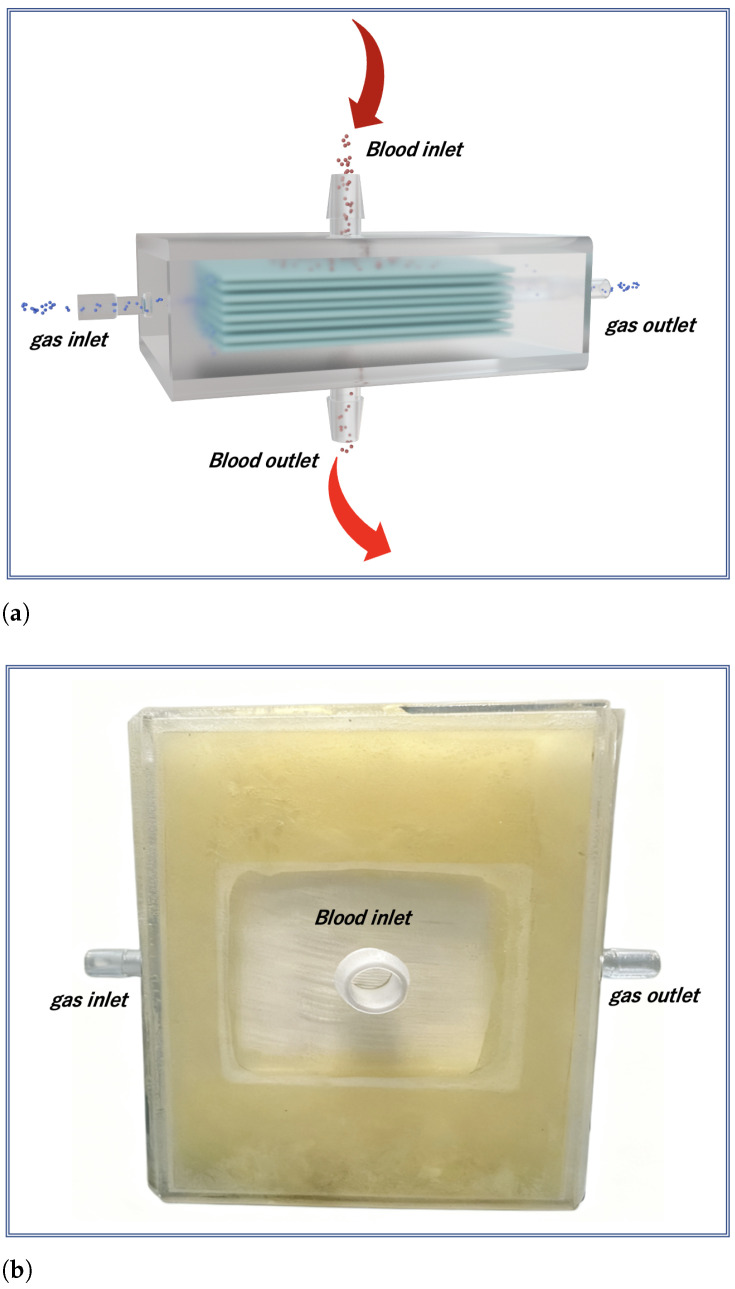
(**a**) Schematic diagram and (**b**) photograph of the membrane oxygenator. Its orthogonal blood-gas flow design simplifies the flow path. This minimized geometric complexity creates a well-controlled environment to isolate the effect of dynamic flow profile regulation on oxygenation performance.

The finalized stacked-plate oxygenator has a fiber bundle volume of 50 mm × 50 mm × 17 mm with a 7 mm blood chamber thickness. It utilizes poly-methylpentene (PMP) fibers of 380 μm external diameter, with a membrane porosity of 0.43, resulting effective membrane area for gas exchange is approximately 0.2 $m^{2}$. The inlet and outlet for the blood are strategically positioned at the geometric center of the fiber membrane, thus ensuring a more uniform flow distribution throughout the blood chamber.The structural design of the oxygenator is shown in Figure 1a. Its internal blood flow field is simplified to reduce the passive mixing effects caused by complex geometries, thereby enabling a more precise evaluation of the impact of flow regulation. Simultaneously, the blood flow direction is completely orthogonal to the gas diffusion direction, which optimizes the mass transfer efficiency between the gas and liquid phases. The overall appearance of the oxygenator is shown in Figure 1b.

### 2.2. Structural Design of Deep Reinforcement Learning Systems

This study employs a DRL approach to identify the optimal flow profile, a process in which an agent learns the optimal policy through interactions with its environment. DRL is particularly suitable for the flow profile optimization problem addressed here, as it can handle continuous action spaces, complex nonlinear dynamics, and multi-objective trade-offs. Its key advantage lies in its ability to learn near-optimal policies directly from data without relying on a precise analytical model.

**Figure 2:**
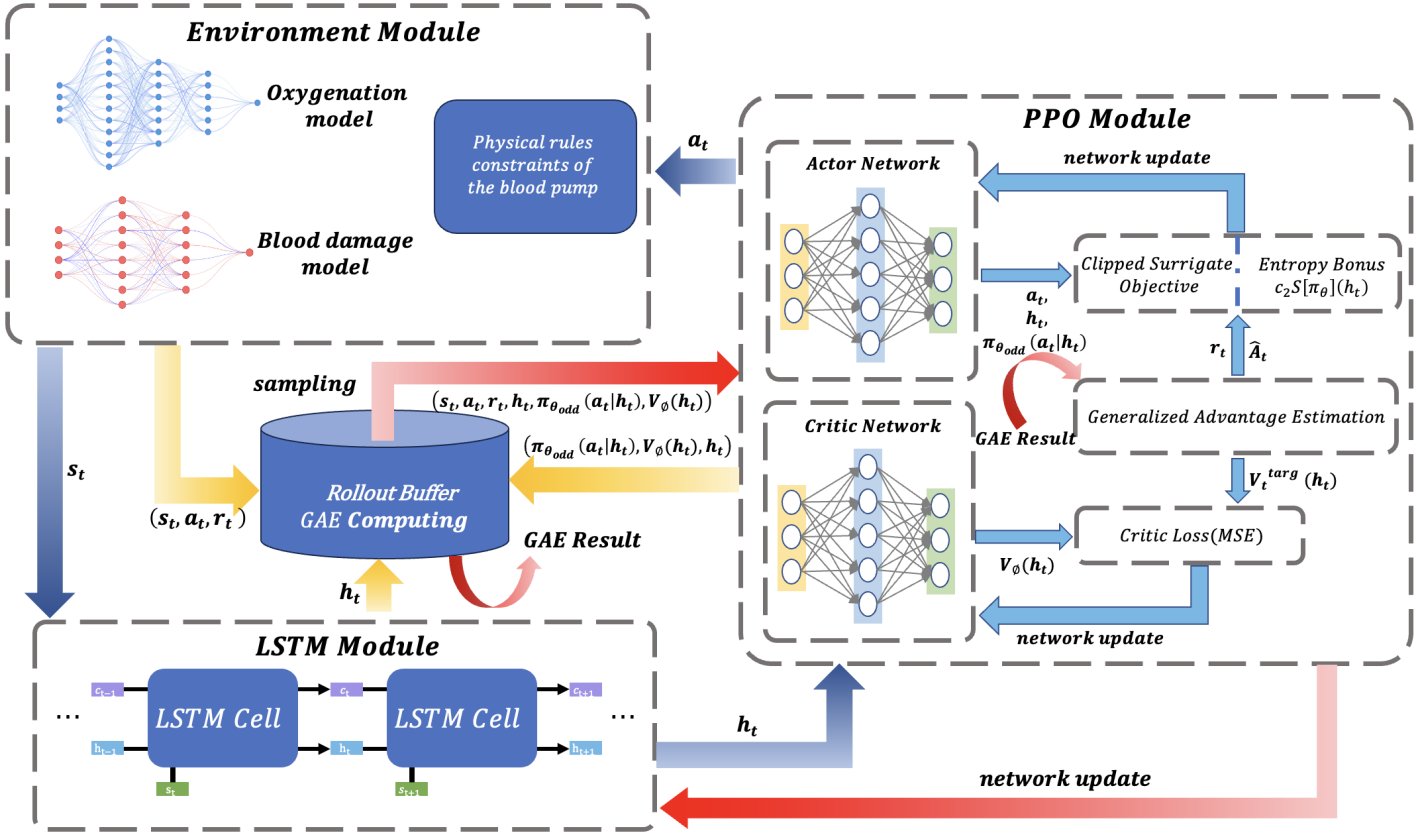
This system diagram illustrates the overall architecture of a deep reinforcement learning (DRL) framework for blood flow profile optimization. The dashed boxes clearly separate four core modules: the Environment Module, LSTM Module, PPO Module, and Rollout Buffer. Arrows indicate the direction of data flow, including the transmission paths of states, actions, rewards, and hidden states.

This system diagram illustrates the overall architecture of a DRL framework, which is shown in Figure 2, aiming to optimize flow profiles through multi-module collaboration. The diagram clearly delineates four core modules using dashed boxes: the Environment Module simulates the physical constraints of the blood pump, oxygenation processes, and blood damage mechanisms, generating the state $s_{t}$ and reward signal $r_{t}$; the Long Short-Term Memory (LSTM) Module extracts the hidden state $h_{t}$ through temporal units to capture dynamic system characteristics; the Proximal Policy Optimization (PPO) Module incorporates an Actor–Critic network to generate the policy $\pi_{\theta }\left(a_{t}|h_{t}\right)$ and evaluate the state value $V_{\theta }\left(h_{t}\right)$; and the Rollout Buffer stores interaction data tuples and integrates Generalized Advantage Estimation (GAE) computations to enhance training efficiency.

The Environment Simulation Module encapsulates key physical and physiological models of the oxygenation process, including the gas transfer model, blood damage model, and the physical constraints of the blood pump. At each time step *t*, the agent generates a control action $a_{t}$ based on the current state $s_{t}$. The environment then returns the next state $s_{t+1}$ and a reward signal $r_{t}$. The reward function is designed to co-optimize oxygenation efficiency, suppress blood damage, and satisfy the pump’s physical operational constraints.

The state $s_{t}$ is fed into the LSTM module, where temporal features are extracted through cascaded LSTM units, outputting a hidden state $h_{t}$. This hidden state is subsequently passed to the PPO module. The Actor network generates the policy $\pi_{\theta }\left(a_{t}|h_{t}\right)$, while the Critic network estimates the state value $V_{\theta }\left(h_{t}\right)$. The data tuples generated from the agent–environment interaction, $\left(s_{t},a_{t},r_{t},h_{t},\pi_{\theta_{\mathrm{old}}}\left(a_{t}|h_{t}\right),V_{\theta }\left(h_{t}\right)\right)$, are stored in an experience replay buffer.

By adapting the methodology established by Liu et al. [37], a complete computational fluid dynamics (CFD) model is developed in this study to simulate blood oxygenation kinetics and damage mechanisms, tailored to the structural parameters of a self-made oxygenator. Specifically, the fiber bundle region is represented as a porous medium following the approach introduced by Zhang et al. [38], wherein the loss coefficients of the porous media model—including the viscous and inertial loss coefficients—are calculated directly from the physical geometric parameters of the fibers, namely the fiber diameter and porosity. This parameterization enables the porous media model to accurately reflect the flow resistance characteristics of the actual fiber bundle. In our simulations, we adopted this well-established method, determining the model coefficients based on measured diameter and porosity values of the fiber bundle, thereby ensuring a physically consistent representation of the real oxygenator structure. Clear boundary conditions were defined in the CFD model to accurately represent the actual operating state of the oxygenator: the inlet was set as a velocity inlet boundary using a formula-defined pulsatile flow waveform to simulate clinical pulsatile blood flow; the outlet was set as a zero-pressure outlet boundary; and the oxygenator wall employed no-slip and zero-flux boundary conditions. Furthermore, a user-defined function was applied to the hollow fiber region to incorporate a mass source term characterizing the oxygen transfer process across the membrane. Since pulsatile blood flow constitutes an unsteady boundary condition, the model was solved using a transient approach.

The resulting framework integrates a gas transfer model  [38] with a blood damage power-law relation [39]:

(1) $$\left(\Delta \mathrm{Hb}/\mathrm{Hb}\right)\left(\%\right)=C\tau^{b}t_{\exp}^{a}$$

where ΔHb denotes the increase in free hemoglobin concentration due to red blood cell damage, Hb represents the total hemoglobin concentration, τ denotes the shear stress, $t_{\exp}$ represents the exposure time, and the parameters *C*, *a*, *b* are referenced from [40]. The hollow fiber bundle is simplified as a porous medium using the Ergun equation to estimate flow resistance.

It is widely recognized that high-fidelity computational fluid dynamics (CFD) simulations incur substantial computational costs [41,42,43]. Directly embedding such an expensive solver within a reinforcement learning framework, which requires numerous iterations, would lead to prohibitively high computational expenses and severely constrain training efficiency. To overcome the computational limitations, this study adopts a data-driven surrogate modeling strategy inspired by [44]. First, a high-fidelity dataset was generated by employing the aforementioned CFD model to simulate the device’s performance under a range of physiological flow profiles. These flow profiles, discretized at a time interval of 0.01 seconds, included a variety of flow profile such as sine waves with varying amplitudes and frequencies, pulmonary artery flow profiles obtained from [45], composite Gaussian profiles, and Gaussian-exponential pulse profiles, among others. For each flow profile, once the CFD simulation reached steady state, the corresponding outputs are the oxygen partial pressure and oxygen saturation before and after oxygenation and hemolysis index at each time point.

The data-driven model comprises two separate fully connected feedforward neural networks (FFNNs) designed to predict oxygen transfer rate and hemolysis index, respectively. The input features for both networks consist of the instantaneous flow rate and its derived properties—namely the first derivative, second derivative, and curvature of the flow profile. The inclusion of curvature, which possesses well-defined geometric and physical implications, allows the model to explicitly leverage established mathematical relationships rather than relearning them, thereby accelerating convergence. The FFNN for oxygen transfer rate prediction adopts a three-hidden-layer architecture (1024, 512, and 64 neurons) with a single output neuron, which is designed to capture the complex nonlinear dynamics inherent in the oxygenation process. In contrast, the hemolysis prediction network employs a simpler two-hidden-layer structure (64 and 16 neurons) with one output, reflecting the mechanical blood damage.

The dataset was partitioned into training, validation, and test sets with a 70%:15%:15% ratio. Model training employed the AdamW optimizer (learning rate 0.0001, weight decay 0.01) to minimize mean squared error loss, incorporating early stopping to prevent overfitting. Performance results are summarized in Table 1. The accuracy values indicate the proportion of predictions that fall within the specified relative error thresholds, meaning these predictions are considered accurate.

This data-driven surrogate model significantly improved computational efficiency while maintaining prediction accuracy, establishing an efficient and reliable interactive environment for subsequent deep reinforcement learning training. It should be noted, however, that although the surrogate model achieves high prediction accuracy, the error propagation effects during its iterative integration within the DRL optimization process have not been systematically quantified. Addressing this limitation represents an important direction for future research.

After completing the interactive environment modeling, a multi-objective reward function must be designed to systematically guide the model’s learning process. The core function of this reward mechanism is to establish an effective trade-off among competing objectives—enhancing gas exchange efficiency, suppressing blood damage, and satisfying physical constraints—by converting complex performance metrics into intelligible reward signals for the agent. This approach drives the exploration of flow profiles strategies that achieve high performance, low risk, and physical feasibility.The expression of the reward function is as shown in (2).

(2) $$r_{t}=\beta_{o}\cdot R_{o}+\beta_{d}\cdot R_{d}+\lambda_{a}\cdot P_{a}+\lambda_{s}\cdot P_{s}+\lambda_{e}\cdot P_{e}$$

The term $R_{o}=\left|\frac{\eta_{t}}{\eta_{\max}}\right|$ corresponds to the oxygenation reward, where $\eta_{t}$ denotes the real-time oxygen transfer rate and $\eta_{\max}$ represents the theoretical maximum transfer rate, while $R_{d}=-\left|\kappa \cdot D_{t}\right|$ acts as a blood damage penalty, with $D_{t}$ quantifying the real-time hemolysis prediction and κ serving as a normalization coefficient. For dynamic constraints, $P_{a}=-\left|{\bar{Q}}_{t}-Q_{tar}\right|$ penalizes deviations from the target flow, where ${\bar{Q}}_{t}$ is the sliding-window flow mean and $Q_{tar}$ is the desired flow rate; $P_{s}=-\frac{1}{W}\sum_{k=t-W+1}^{t-1}\left|a_{k}\right|$ enforces smoothness by averaging flow acceleration $a_{k}$ over a window of size *W*; and $P_{e}=-\left|Q_{T}\right|\cdot 1_{|Q_{T}|>\tau }$ imposes a terminal flow constraint, where $Q_{T}$ is the final flow rate and τ is a tolerance threshold. The weighting coefficients $\beta_{o}$, $\beta_{d}$, $\lambda_{a}$, $\lambda_{s}$, and $\lambda_{e}$ independently regulate the influence of each term, ensuring a balance between performance optimization and operational feasibility.

### 2.3. Code Implementation and Training Protocol

To obtain an optimal blood flow profile, this study develops a deep reinforcement learning environment based on the OpenAI Gym framework, utilizing a Recurrent Proximal Policy Optimization (Recurrent PPO) algorithm with a shared-feature temporal decision model. The model integrates multidimensional dynamic observations—denoted as (3), including real-time flow rate, a five-step historical flow sequence, normalized step-based time progression, and cumulative remaining flow demand—via a 256-unit LSTM network. This architecture captures long-term temporal dependencies and extracts unified features for both Actor and Critic networks, thereby facilitating coordinated policy and value function updates through a synchronized optimization process where model weights are concurrently updated via a combined loss function integrating policy gradients with value estimation errors.

The agent is designed to sequentially construct a full pulsatile flow profile over a fixed-duration cycle. At each 4-ms time step—a interval determined by the pump’s physical actuation capability—the agent outputs a normalized control signal $a_{t}\in \left[-1,1\right]$, which corresponds to a desired flow change. This command is scaled by the pump’s maximum permissible stepwise flow variation of 0.4 L/min, yielding the actual flow adjustment $\Delta Q_{t}$. The new flow rate $Q_{t}$ is then computed based on the previous value $Q_{t-1}$ and strictly clipped to the operating bounds of −0.5 to 5.0 L/min, as defined by the pump’s mechanical and safety limits. This stepwise process repeats over 200 consecutive intervals, progressively building a physiologically plausible and physically realizable flow trajectory in which each point complies with the pump’s inherent step-change and absolute flow constraints.

Training employs a phased curriculum strategy that dynamically adjusts multi-objective reward weights and exploration parameters: an entropy coefficient of 0.05 promotes broad exploration initially (0–800 k steps), 0.03 balances exploration and exploitation intermediately (800 k–1.2 M steps), and 0.01 enhances determinism terminally (>1.2 M steps). The PPO algorithm ensures stability via a clipped surrogate objective (4), while normalization and physiological constraints maintain numerical stability and flow profile feasibility, collectively enabling generation of pulsatile profiles with high oxygenation efficiency and minimal hemolytic damage.

(3) $$s_{t}=\left[Q_{t},Q_{t-n:t},T_{progress},D_{remain}\right]$$

where $Q_{t}$ denotes the instantaneous flow rate at the current time step, $Q_{t-n:t}$ represents the historical flow sequence within an *n*-step sliding window (n=5), $T_{\mathrm{progress}}$ indicates the time step progression, and $D_{\mathrm{remain}}$ corresponds to the remaining flow demand.

(4) $$L^{\mathrm{CLIP}}=E_{t}\left[\min\left(r_{t}{\hat{A}}_{t},\;\mathrm{clip}\left(r_{t},1-\epsilon ,1+\epsilon \right){\hat{A}}_{t}\right)\right]$$

where $r_{t}=\frac{\pi_{\theta }\left(a_{t}|h_{t}\right)}{\pi_{\theta_{\mathrm{old}}}\left(a_{t}|h_{t}\right)}$ represents the probability ratio comparing the new policy $\pi_{\theta }$ to the old policy $\pi_{\theta_{\mathrm{old}}}$, and $h_{t}$ is the latent state feature (derived from an LSTM network); ${\hat{A}}_{t}$ denotes the generalized advantage estimate, which quantifies the relative benefit of an action by combining multi-step temporal-difference errors; and ϵ is the clipping threshold that bounds the ratio within [1−ϵ,1+ϵ] to prevent excessive policy changes. This objective function operates over expected trajectories to balance exploration and exploitation, effectively maintaining training stability while optimizing for complex tasks such as pulsatile flow control in membrane oxygenators, as detailed in Appendix A and [46].

### 2.4. Training Result

**Figure 3:**
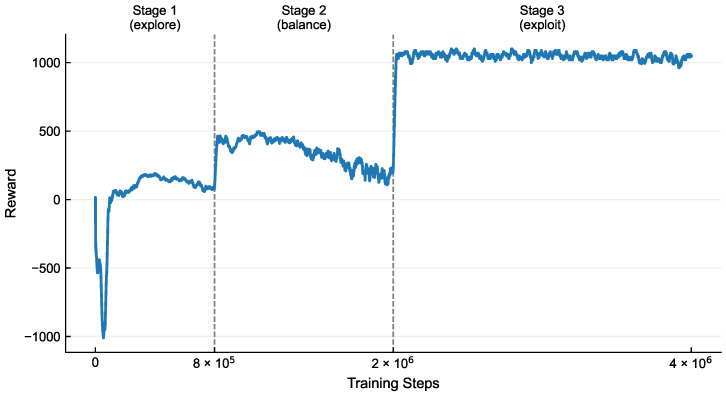
The reward curve demonstrates three distinct phases: an initial explore stage characterized by high volatility and low average rewards under high entropy coefficients; a transitional balance phase marked by steady ascent with reduced fluctuations as the agent shifts from exploration to exploitation; and a final exploit stage evidenced by convergence to a high-value plateau, reflecting refined policy determinism.

The dynamic variation of the reward values throughout the training process is shown in Figure 3, and its evolution clearly reflects the learning trajectory of the agent under the guidance of the phased training strategy. During the initial training phase, the reward curve exhibits significant fluctuations and a relatively low mean value, which aligns with the strategy of using a high entropy coefficient (0.05) to encourage extensive exploration. In this stage, the agent actively explores the environment by trying diverse actions, accumulating essential experience for subsequent optimization. In the intermediate phase, the reward values demonstrate a steady upward trend with noticeably reduced fluctuations, indicating that the agent transitions from random exploration to targeted exploitation, consistently selecting high-reward actions. In the final phase, the reward values further converge to a high and stable plateau with minimal oscillations, reflecting that the policy, after fine-tuning with a low entropy coefficient (0.01), has achieved high determinism and can generate stable and high-performance pulsatile flow profiles under complex multi-objective constraints.

**Figure 4:**
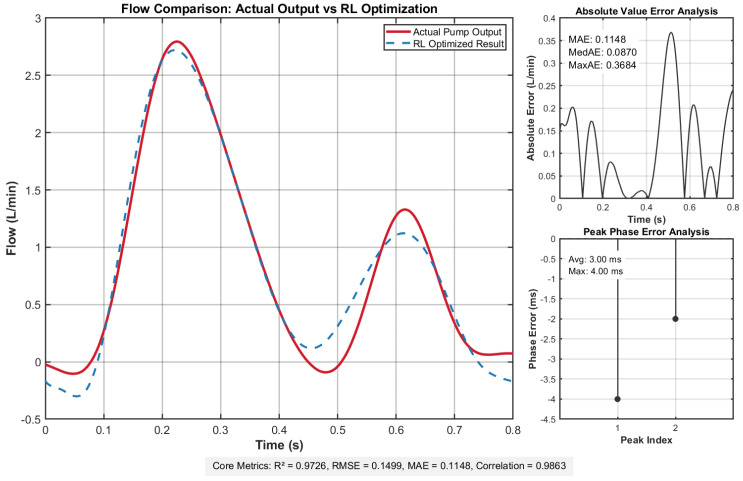
Flow comparison results demonstrate excellent agreement between actual and target flow profiles, with minimal deviation evidenced by low error metrics: root mean square error (RMSE) = 0.1499, mean absolute error (MAE) = 0.1148, and uniform error distribution. Temporal alignment is precise, showing an average delay of 3.00 ms and maximum delay of 4.00 ms. Statistical validation confirms strong consistency, with a coefficient of determination (R) of 0.9726 and correlation coefficient of 0.9863, underscoring robust flow profile replication fidelity.

Figure 4 compares the reinforcement learning-optimized flow profile with the pump system’s actual output, demonstrating their close alignment in morphology, amplitude, and dynamic response. The inset quantitatively analyzes the instantaneous flow error distribution and inter-peak timing differences.

These metrics collectively affirm that the actual output flow achieves remarkable congruence with the deep reinforcement learning-optimized target curve, validating the system’s capability to faithfully replicate the desired flow dynamics. This close alignment ensures that the pump system meets stringent experimental requirements for both precision and operational reliability.

## 3. Experiment

**Figure 5:**
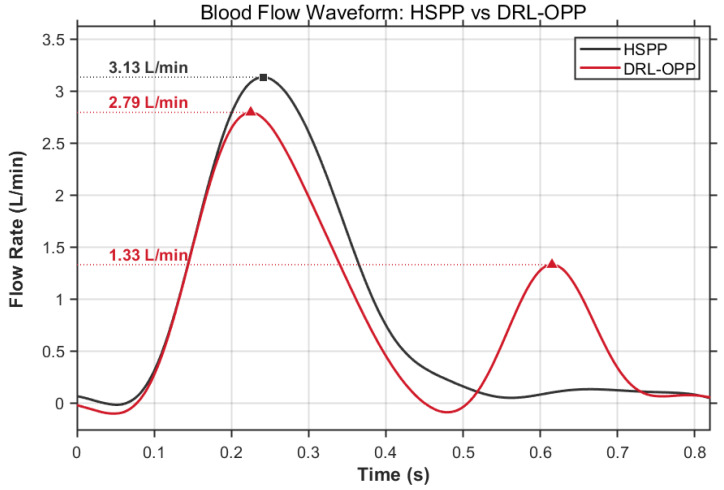
Comparative Analysis of Blood Flow Profiles: Hemodynamic Performance of HSPP vs. DRL-OPP. The HSPP reached a peak flow rate of 3.13 L/min around 0.24 s, whereas the DRL-OPP reached a peak flow rate of 2.79 L/min around 0.22 s. Notably, the DRL-OPP exhibited a distinct secondary peak (1.33 L/min) around 0.62 s.

This study aims to systematically investigate the effects of different profiles generated by a pulsatile pump on blood oxygenation efficiency and cellular damage characteristics. The experiments were conducted at Yinshe (Shanghai) Biotechnology Co., Ltd. (Shanghai, China), with all blood sample collections complying with relevant laws and regulations and approved by the company’s ethics committee (YS202509-132B). All samples were properly disposed of after the experiments. The study utilized a ultrasonic motor-driven pump and stacked membrane oxygenators, where the pulsatile pump, featuring high precision, rapid response, and power-off self-locking, accurately output different pulsatile flow profile. Experiments were performed under strictly controlled parameters: the average blood flow rate was maintained constant at 0.9 L/min, and the pulsation period was set to 0.8 s. The 0.9 L/min blood flow rate was selected based on three principal considerations: it lies within the typical clinical range for adult partial assistance and pediatric ECMO support (consistent with the operational specifications of commercial infant oxygenators such as the AMG PMP and Trilly models), aligns with the optimal operating range of the experimental blood pump, and corresponds appropriately to the membrane area of our custom stacked-plate oxygenator. The pulsation period was primarily determined by the average flow rate requirement and the specific characteristics of the pump. The oxygenation performance and blood damage evaluation of two distinct flow profiles—a standard half-sine pulse profile (HSPP) and a deep reinforcement learning-optimized pulsatile profile (DRL-OPP), as illustrated in Figure 5—were evaluated against the ISO 7199:2016 standard. The assessment specifically quantified hemolytic potential via plasma free hemoglobin concentration and coagulation activation via thrombin–antithrombin complex (TAT) levels.

The experimental procedure was divided into two parts: the measurement of the oxygen transfer rate and the assessment of hemolysis and coagulation activation, to comprehensively quantify the physiological compatibility and performance differences of the flow profiles. To ensure consistency, all blood used in the same set of experiments was sourced from a single donor pig.

**Figure 6:**
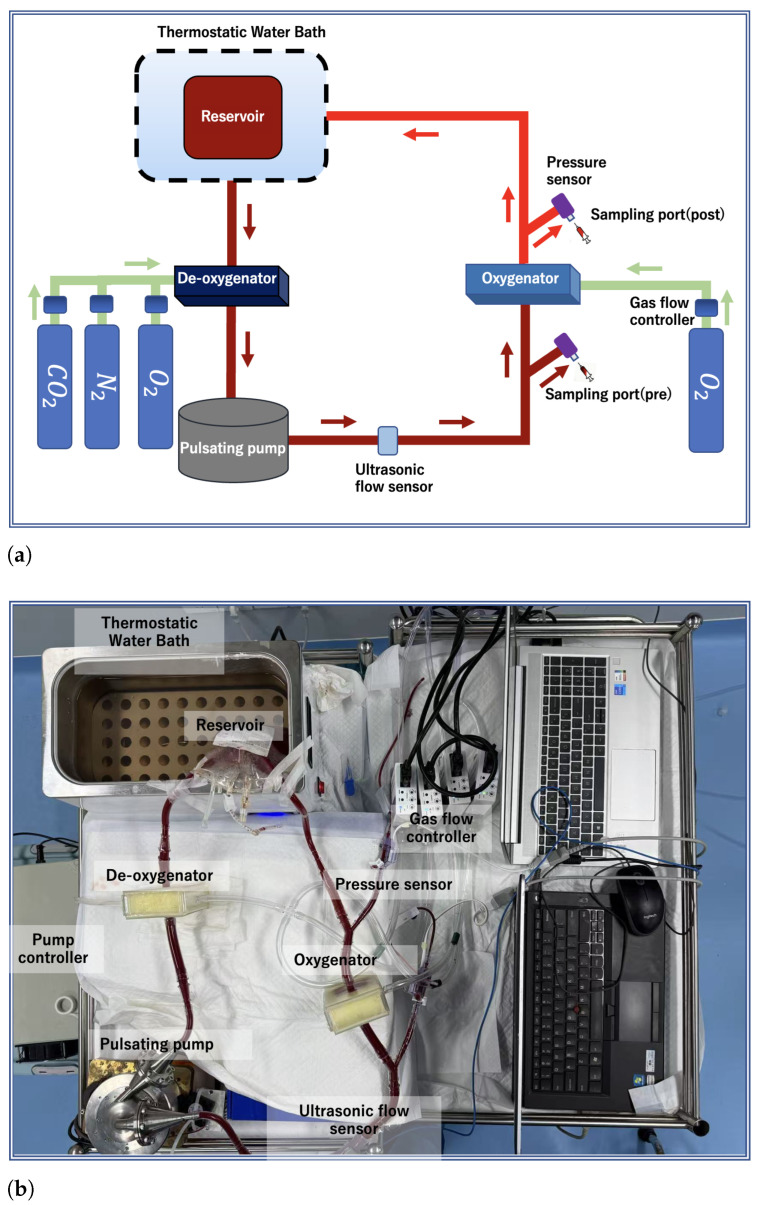
Oxygenator experimental setup: (**a**) schematic diagram of the circuit; (**b**) physical photograph of the system.

The extracorporeal circulation circuit used in the oxygenation experiment primarily consisted of a pulsatile pump [47] (a pulsatile blood pump with two bioprosthetic valves (BalMedic, Beijing, China) was used to generate pulsatile flow), several laboratory-developed oxygenators, a constant-temperature water bath (Jiu’an, Huzhou, China), and a blood storage bag (Nige’er, Chengdu, China). All components were connected using polyvinyl chloride (PVC) tubing (3/8-inch PVC tubes, Weihai, China) and connectors. The specific circuit layout is illustrated in Figure 6. Prior to use, the entire circuit was pre-filled and degassed with a 0.9% sodium chloride solution. Fresh porcine blood was injected into the storage bag and delivered through the oxygenator via the pulsatile pump. A flow probe (an ultrasonic flow sensor (Transonic System, Ithaca, NY, USA)) was employed to monitor the blood flow rate, enabling precise speed control of the ultrasonic motor.

### 3.1. Blood Oxygenation Experiment

During oxygenation, the gas flow rate of the oxygenator was regulated using a flow sensor (Biosflow FDC-300 Series) to maintain a gas-to-blood flow ratio of 2:9. To create a deoxygenation environment and achieve dynamic gas balance within the circuit, nitrogen ($N_{2}$) and carbon dioxide (${\mathrm{CO}}_{2}$) were supplied to the deoxygenation oxygenator at flow rates of 2 L/min and 0.24 L/min, respectively. Blood samples were collected from sampling ports located before and after the oxygenator. Prior to each formal sampling, 1 mL of blood was drawn to flush out any residual stagnant blood in the ports. For each flow profile condition, the experiment was independently repeated three times. In each experimental replicate, after the system re-established equilibrium, 1 mL blood samples were collected from both the pre- and post-oxygenator positions. The collected samples were immediately analyzed using an EDAN i15 bedside dry blood gas analyzer. The oxygen ($O_{2}$) transfer rates per liter of blood flow were subsequently calculated according to Equations (5) and (6).

(5) $$C_{O_{2}}=13.4\times \mathrm{Hb}\times \frac{{\mathrm{SO}}_{2}\%}{100}+0.0314\times pO_{2}$$

(6) $$O_{2\mathrm{Transfer}\;\mathrm{Rate}}=\left(C_{O_{2}\mathrm{post}}-C_{O_{2}\mathrm{pre}}\right)$$

where $C_{O_{2}}$ is the total oxygen concentration in blood, Hb is the hemoglobin concentration in blood, $SO_{2}\%$ is the hemoglobin oxygen saturation, $pO_{2}$ is the oxygen partial pressure in the blood, $C_{O_{2}\mathrm{post}}$ is the oxygen concentration at the blood outlet, and $C_{O_{2}\mathrm{pre}}$ is the oxygen concentration at the blood inlet.

### 3.2. Hemolysis and Coagulation Activation Assessment Experiment

**Figure A1:**
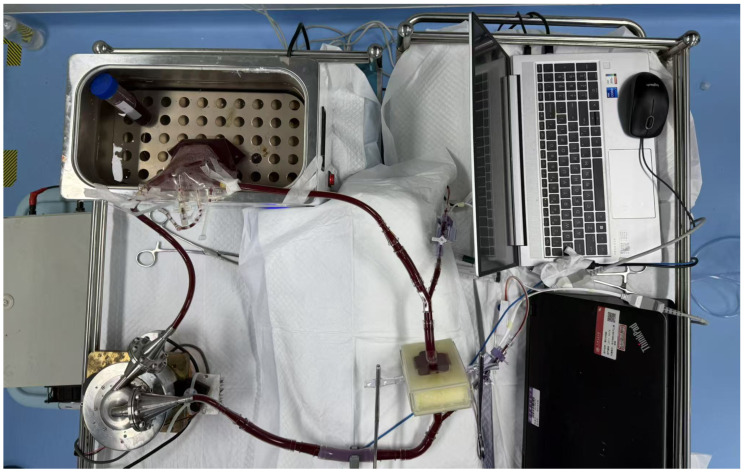
Photograph of the experimental setup for hemocompatibility assessment. The circuit configuration excluded the deoxygenation oxygenator and its gas supply, and the same circuit was used to evaluate both the HSPP and DRL-OPP profiles under identical hardware conditions.Blood samples were collected at 0, 2, and 4 hours during circulation. Concentrations of plasma free hemoglobin (PFH) and thrombin–antithrombin complex (TAT)—key indicators of overall circuit-induced hemolysis and coagulation activation—were determined using commercial ELISA kits (96-well plate format) by Omais (Shanghai) Biotechnology Co., Ltd. (Shanghai, China), in accordance with standardized protocols.

In the hemolysis and coagulation activation assessment experiment, the circuit was simplified by removing the deoxygenation oxygenator and its associated gas supply equipment, which is shown as Figure A1. At the beginning of the experiment (0 h), 30 mL of blood was drawn as a static control group and placed statically in a constant-temperature water bath. The remaining blood was retained in the storage bag for the circulation experiment. The circulation test lasted for 4 h, with blood samples collected every two hours. The concentrations of plasma free hemoglobin (PFH) and thrombin–antithrombin complex (TAT) were measured from these samples. The normalized index of hemolysis (NIH) was then calculated based on the PFH data according to the ASTM F1841-19 standard [48]. In this study, the differences in hemolysis and coagulation activation between the DRL-OPP and the HSPP were the primary focus of the hemocompatibility assessment, which was conducted with three independent replicates.

### 3.3. Statistical Analysis

Statistical analysis was performed using NumPy (version 1.26.4) and SciPy (version 1.13.1). Descriptive statistics were conducted by calculating the standard error of the mean (SEM) for oxygenation efficiency across experimental groups. An independent samples *t*-test was employed to assess the significance of differences in mean gas transfer rates between the HSPP and DRL-OPP groups. A *p*-value < 0.05 was considered statistically significant.

## 4. Results and Discussion

### 4.1. Oxygen Transfer

**Figure 7:**
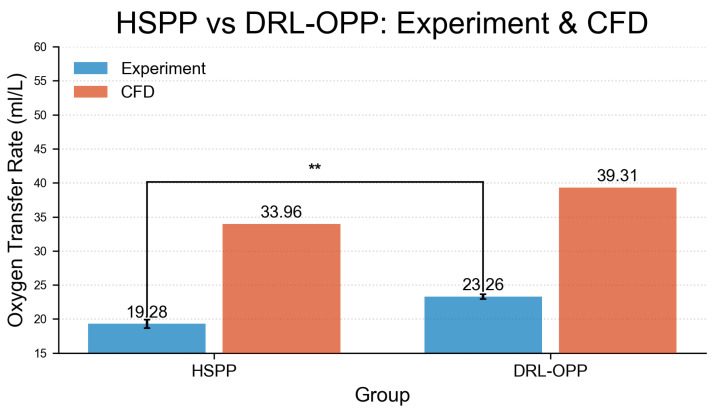
Comparison of oxygen transfer rate between HSPP and DRL-OPP groups under experimental and computational fluid dynamics (CFD) conditions. A highly significant difference (** p<0.01) was observed between CFD and experimental values in the HSPP group. All data points represent mean values, with error bars indicating the standard error of the mean (SEM).

The experimental measurements and CFD simulation results for oxygen transfer rate (OTR) are presented in Figure 7, revealing significant differences between profiles. Under the same experimental conditions, the HSPP group achieved an OTR of 19.28 mL/L, while the DRL-OPP group increased to 23.26 mL/L—a 20.64% improvement over HSPP. In CFD simulations, the HSPP group yielded an OTR of 33.96 mL/L, with the DRL-OPP group significantly rising to 39.31 mL/L, representing a 15.75% enhancement. Despite discrepancies in absolute OTR values—primarily stemming from the well-understood limitations of the porous media model in resolving local flow structures, coupled with inherent numerical errors and parameter uncertainties—both experimental and CFD results are in consensus that the DRL-OPP achieves a substantial improvement in oxygen transfer over the HSPP profile.

The performance improvement of DRL-OPP may be attributed to its unique hemodynamic characteristics. Under identical pulsation rates and average flow conditions, DRL-OPP induces stronger periodic vibrations that amplify turbulent components in the blood flow. This enhanced turbulence can effectively reduce the thickness of the laminar boundary layer at the membrane surface, thereby promoting relative motion between red blood cells and the oxygenator membrane and improving blood mixing. To clarify the relative contributions of this enhanced mixing versus other factors such as residence time, targeted quantitative studies employing specific techniques would be required in future research.

### 4.2. Hemolysis and Coagulation Activation

**Figure 8:**
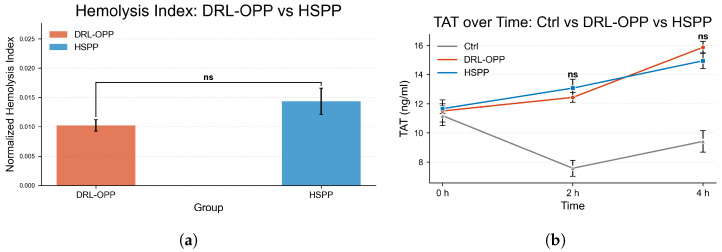
(**a**) Comparison of Normalized Index of Hemolysis (NIH) and (**b**) Thrombin–Antithrombin complex (TAT) between HSPP and DRL-OPP. Although numerical differences were observed, no statistically significant difference was found between the HSPP and DRL-OPP groups (*p* > 0.05) for both hemocompatibility metrics. Error bars represent the standard error of the mean (SEM).

Figure 8a compares the normalized index of hemolysis between DRL-OPP and HSPP. Although the HSPP group showed numerically higher values, the difference was not statistically significant (labeled as “ns”).

Figure 8b illustrates the temporal evolution of thrombin–antithrombin complex (TAT) concentrations across the control (Ctrl), DRL-OPP, and HSPP groups. At the initial phase (0 h), TAT levels were closely aligned among all three groups, confirming consistent baseline conditions. As circulation time progressed to 2 h, both the DRL-OPP and HSPP groups exhibited a marked elevation in TAT concentrations, significantly surpassing those of the control group. Notably, no statistically significant difference (labeled as “ns”) was detected between the DRL-OPP and HSPP groups at this stage. By the 4 h time point, TAT levels in the DRL-OPP and HSPP groups further increased and remained substantially higher than the control, yet again, no significant divergence emerged between the two experimental flow profiles. These findings demonstrate that while prolonged extracorporeal circulation intensified coagulation activation, the DRL-optimized and conventional pulsatile profiles exerted statistically indistinguishable effects on thrombogenic potential. Normalized hemolysis index and TAT values are detailed in Table 2 and Table 3.

The primary motivation for this experimental study stems from the hemodynamic advantages of pulsatile flow over constant-flow perfusion, particularly in enhancing microcirculatory perfusion and preventing thrombosis. Building on this premise, the present work focuses on the pulsatile modality per se, adopting a technical pathway dedicated to optimizing its flow waveform in order to fully exploit its inherent benefits. Guided by this focus, the experimental results demonstrate that the deep reinforcement learning-optimized profile (DRL-OPP) significantly enhances the oxygen transfer rate compared to conventional sinusoidal pulsatility, without inducing a significant increase in red blood cell hemolysis or coagulation activation. The optimized profile exhibits a distinct dual-peak characteristic within a single cycle. Compared to the conventional sinusoidal pulsatile flow, this configuration intensifies the perturbation of the blood flow boundary layer, thereby promoting gas exchange. Furthermore, the lower amplitude of its primary peak effectively reduces the overall shear stress imposed on the blood. These combined hydrodynamic effects underpin the maintained hemocompatibility, as evidenced by the nonsignificant changes in hemolytic and coagulation markers. The synergy between enhanced boundary layer disruption and controlled shear stress generation positions DRL-OPP as a potentially promising strategy for achieving superior gas exchange while preserving hemocompatibility in extracorporeal systems.

Despite these promising results, it is important to acknowledge the limitations of this study. First, the current validation is limited to a specific oxygenator configuration. Although the proposed DRL framework’s core strength is the flexibility of its simulation environment—enabling adaptation to different geometries—this study does not include experimental testing on oxygenators of differing sizes or configurations. Furthermore, the performance assessment was conducted exclusively between different pulsatile flow patterns. A direct, controlled comparison with a conventional constant blood flow under identical conditions was not performed, which limits the evaluation of the DRL-OPP’s relative advantage over standard clinical operation. These aspects warrant further investigation in future studies.

## 5. Conclusions

This study, conducted in compliance with the ISO 7199:2016 standard, establishes a comprehensive in vitro evaluation system integrating gas exchange and blood damage assessment to comparatively analyze the effects of the half-sine pulse profile (HSPP) and the deep reinforcement learning-optimized pulsatile profile (DRL-OPP) on oxygen transfer rate and hemocompatibility. The experiments were performed under rigorously controlled conditions, with a consistent average flow rate of 0.9 L/min and a pulsation frequency of 1.25 Hz across all experimental groups, ensuring that flow profile modulation served as the sole independent variable. Results demonstrate that the DRL-OPP significantly enhances oxygen transfer efficiency by 20.64% compared to the HSPP, while no statistically significant differences were observed in the normalized index of hemolysis or thrombin–antithrombin complex (TAT) levels between the two flow profile groups. This indicates that the optimized flow profile improves gas exchange performance without exacerbating hemolytic or coagulative activation.While this study focused on pulsatile flow profiles, future work will include comparisons with constant flow to provide broader context.

This study demonstrates that intelligent optimization of the pulsatile flow waveform in a pulsatile artificial lung system can enhance gas exchange efficiency without compromising the intrinsic design advantages of current oxygenators—namely, low priming volume and compact membrane area. This approach thereby provides a viable technical pathway for the development of next-generation portable extracorporeal life support systems based on pulsatile flow. The core challenge for such pulsatile-flow-based portable devices lies in achieving sufficient oxygenation within a constrained volume. Unlike conventional approaches that rely on enlarging membrane surface area or modifying materials, this study employs deep reinforcement learning to optimize the pulsatile flow waveform, effectively improving oxygen transfer rates without altering the oxygenator hardware or introducing additional moving components. This enables future pulsatile pump-lung devices to meet performance targets in more compact designs and at lower inspired oxygen concentrations (FiO_2_). The decreased (FiO_2_) requirement mitigates long-term oxygen toxicity risks while easing gas supply demands—a particular advantage for portable systems operating with limited oxygen sources. Consequently, this approach directly promotes device miniaturization, enhances operational safety, and improves overall system sustainability in the design of wearable, pulsatile-flow artificial lungs.

## Figures and Tables

**Table 1:** Neural Network Model Performance Comparison (Relative Error Threshold).

Model Type	Accuracy at Different Relative Error Thresholds				
	1%	2%	3%	4%	5%
Oxygen Transfer	0.62	0.87	0.95	0.97	0.98
Blood Damage	0.70	0.92	0.96	0.97	0.97

Note: Accuracy values reflect the percentage of predictions deemed accurate by falling within the relative error thresholds.

**Table 2:** Comparison of Normalized Hemolysis Index (NIH) Between DRL-OPP and HSPP Flow Profiles.

Group	NIH (Normalized Index)	*p*-Value (DRL-OPP vs. HSPP)
DRL-OPP	0.01023 ± 0.00099	0.1648 (ns)
HSPP	0.01435 ± 0.00222	

Note: Values represent mean ± SD. ns = not significant (*p* > 0.05).

**Table 3:** Temporal Comparison of Thrombin–Antithrombin Complex (TAT) Levels.

Group	0 h TAT (ng/mL)	2 h TAT (ng/mL)	4 h TAT (ng/mL)
Ctrl	11.18 ± 0.67	7.58 ± 0.54	9.42 ± 0.74
DRL-OPP	11.51 ± 0.76	12.44 ± 0.33	15.87 ± 0.41
HSPP	11.66 ± 0.34	13.07 ± 0.61	14.95 ± 0.54

Note: Values represent mean ± SD. (*p* > 0.05).

## Data Availability

The data presented in this study are available on request from the corresponding author due to (ethical reasons).

## Appendix A. Proximal Policy Optimization with LSTM for Blood Flow Profile

**Algorithm 1:** PPO with LSTM Sequence Processing
**Input**: Initial parameters θ, ϕ, LSTM hidden states $h_{0}^{p}$, $h_{0}^{v}$ **Output**: Optimized policy $\pi_{\theta }$ and value function $V_{\phi }$ **for** *iteration = 1 to N* **do** **end**
